# Invalidation of the Transcriptional Modulator of Lipid Metabolism PPARβ/δ in T Cells Prevents Age-Related Alteration of Body Composition and Loss of Endurance Capacity

**DOI:** 10.3389/fphys.2021.587753

**Published:** 2021-03-17

**Authors:** Anne-Sophie Rousseau, Joseph Murdaca, Gwenaëlle Le Menn, Brigitte Sibille, Walter Wahli, Sébastien Le Garf, Giulia Chinetti, Jaap G. Neels, Isabelle Mothe-Satney

**Affiliations:** ^1^Université Côte d’Azur, INSERM, C3M, Nice, France; ^2^Center for Integrative Genomics, Faculty of Biology and Medicine, University of Lausanne, Lausanne, Switzerland; ^3^Lee Kong Chian School of Medicine, Nanyang Technological University, Singapore, Singapore; ^4^Toxalim (Research Centre in Food Toxicology), INRA, Toulouse, France; ^5^Université Côte d’Azur, CHU, INSERM, C3M, Nice, France

**Keywords:** regulatory T cells, skeletal muscle, aging, immunometabolism, physical capacity

## Abstract

Anti-inflammatory regulatory T cells (Tregs) are the most metabolically flexible CD4^+^ T cells by using both glycolysis and fatty acid oxidation (FAO) which allow them to migrate in tissues. With aging, Tregs accumulate in secondary lymphoid organs and are involved in impairment of skeletal muscle (SKM) regeneration and mass maintenance. In this study, we showed that a deletion of a FAO modulator, peroxisome proliferator-activated receptor beta/delta (PPARβ/δ), specifically in T cells (KO-T PPARβ/δ), increased the number of CD4^+^ T cells at day 2 following a cardiotoxin-induced SKM regeneration. Older KO-T PPARβ/δ mice maintained a Tregs prevalence in lymph nodes similar to young mice. Surprisingly, KO-T PPARβ/δ mice were protected from the effects of age on lean and fat mass and endurance capacity. Our results lead us to propose an original potential role of T cell metabolism in the effects of aging on the maintenance of body composition and endurance capacity.

## Introduction

Agonists of the peroxisome proliferator activated receptor beta/delta (PPARβ/δ) have been studied this last decade as “exercise-mimetics” that are susceptible to be therapies for metabolic diseases by increasing skeletal muscle (SKM) fatty acid metabolism (Wall et al., 2016; Fan et al., 2017). The expression level of PPARβ/δ is one of the determinants of its activity (Rousseau et al., 2016) and of its physiological role in the regulation of inflammation (Neels and Grimaldi, 2014). Although PPARβ/δ is highly expressed in SKM, it is broadly expressed in other tissues including lymphoid organs (Girroir et al., 2008) where its activity increased after administration of its specific agonist GW0742 (Le Garf et al., 2019). A critical role of PPARβ/δ in T cell functions has been suggested by studies using whole body PPARβ/δ-deficient mice (Dunn et al., 2010; Kanakasabai et al., 2011; Zhao et al., 2018). However, these studies did not allow determining whether the effects observed on immune dysregulations were directly induced by specific PPARβ/δ absence in T cells. By treating mice with GW0742 or by using a mouse model specifically overexpressing PPARβ/δ in T cells, we showed a drastic decrease in alpha/beta T cells that was accompanied by a characterized thymus involution (Mothe-Satney et al., 2016). This might reflect an adaptation of the energetic metabolic cost of thymus function and resemble, in some aspects, to changes observed with aging (Sato et al., 2017). Immune aging is associated with a decline in T cell activation and proliferation involving alteration of signaling pathways in T cells such as nuclear factor kappa B (NF-κB) (Bektas et al., 2013, 2017) which activity can be regulated by PPARβ/δ (Schnegg et al., 2012). In addition, alteration of lipid metabolism, that determines lipid rafts and fatty acid oxidation (FAO), also contributes to the decline in T cell functions with age (Larbi et al., 2004) and their ability to survive (Yanes et al., 2019). Whether this aging effect involves an increased activation of PPARβ/δ is unknown but overexpression/high activity of PPARβ/δ in T cells in healthy animals could be potentially deleterious for the maintenance of a normal immune function.

We have previously shown that CD4^+^ T cells are able to increase their FAO after *in vitro* treatment with GW0742 (Mothe-Satney et al., 2016). The delineation of the different utilization of metabolic pathways by distinct subsets of T cells (Michalek et al., 2011; Newton et al., 2016) has led to the exciting possibility that PPARβ/δ activation could increase the presence of subsets which are mainly dependent on FAO such as the anti-inflammatory Tregs (Berod et al., 2014; Newton et al., 2016; Cluxton et al., 2019). This hypothesis is supported by our data showing that GW0742 increased the polarization of CD4^+^ Tregs *in vitro* (unpublished) and the prevalence of Tregs in lymph nodes (LNs) during weight loss in trained mice (Le Garf et al., 2019). Lifelong aerobic training has been regarded as a preventive strategy by improving the anti-inflammatory environment and by allowing the maintenance of circulating Tregs with aging (Minuzzi et al., 2019). However, whether these effects are driven by modifications of T cell metabolism is unknown. If improving PPARβ/δ activity could increase a Tregs phenotype, probably by increasing CD4^+^ T cell FAO, it could also induce a metabolic inflexibility that would impair the migration capacity of Tregs in inflamed tissues which has been shown to depend on glycolysis (Kishore et al., 2017). This Tregs migration impairment could be deleterious notably in the context of SKM regeneration and SKM mass maintenance. SKM regeneration is driven during the early stage after injury by an increasing and coordinate infiltration and activity of immune cells (Tidball, 2017). The accumulation of Tregs shown at day 4 post-injury (Burzyn et al., 2013; Schiaffino et al., 2016), dampening inflammation (Panduro et al., 2018), profoundly declined with age paralleling a degradation of repair and regeneration processes (Kuswanto et al., 2016). This could be underpinned by the reduced mobilization of Tregs from the LNs of aged mice and/or their diminished recruitment to injured muscle (Kuswanto et al., 2016). Indeed, Tregs were shown to accumulate in secondary lymphoid organs with aging (Darrigues et al., 2018; Durand et al., 2018).

In this study, we asked the basic question of whether the invalidation of PPARβ/δ specifically in T cells alters the T cell profile in lymphoid organs and peripheral tissues. To do so, we investigated whether the age-related alteration of Tregs population was modified with this specific invalidation and whether this could alter the maintenance of SKM mass and physical capacities.

## Materials and Methods

### Mice

Lck-Cre mice (Cre recombinase under control of the T cell-specific Lck gene promoter) were obtained from Jackson Laboratory [B6.Cg-Tg(Lck-cre)548Jxm/J, stock number 003802]. B6.Ppard^TM 1*Mtz*^ mice (that possess loxP sites up- and down-stream of PPARβ/δ exon 4) were previously generated (Schuler et al., 2006). Both strains are on the C57BL/6J background and were crossed to obtain T cell-specific PPARβ/δ knockout mice (named KO-T PPARβ/δ mice). It has previously been reported that Cre expression in Lck-Cre mice results in off-target effects including a decrease in thymic cellularity (toxic to CD4^+^CD8^+^ cells) (Shi and Petrie, 2012). Therefore, hemizygous Lck-Cre mice were used as controls. We used young (12–16 weeks) and older mice (39–45 weeks) of both sexes. Animals were maintained in a 12-h light, 12-h dark cycle and received food [A04 from UAR (Usine d’Alimentation Rationnelle), Villemoisson sur Orge, France] and water *ad libitum* (agreement number of the animal facility: A 06-088-014). All experimental procedures were conducted according to French legislation and to the EU Directive 2010/63 for animal experiments and were approved by the Institutional Ethic Committee for the Use of Laboratory Animals (CIEPAL-AZUR; N°2018110914193037).

### Acute Muscle Injury

Mouse SKM were injured by injection of 50 μl of cardiotoxin (CTX) from *Naja pallida* at 0.03 mg/ml (L8102, Latoxan, France) in the left *Tibialis Anterior* (TLA), under gas anesthesia (5% Vetflurane). The right TLA (control leg) received 50 μl of saline solution (0.9% NaCl). In order to avoid excessive pain, mice received a subcutaneous injection of buprenorphine (100 μl at 30 μg/ml) 20 min before anesthesia. Before the killing, occurring at days 2 and 4 post-injury by intracardiac puncture, mice received an intraperitoneal injection of ketamine/xylazine (100 and 16 mg/kg, respectively). Different tissues (TLA and other SKM, heart, liver, spleen, lymph nodes, thymus, brown, and white adipose tissues) were harvested, weighed (except lymph nodes), and used for further analyses (see below).

### Physical Test and Body Composition Measurements

Physical tests and body composition measurements were performed 2 days before CTX injection.

### Mice Endurance Evaluation

The endurance of the mice was evaluated using a treadmill running test (five-lane motorized treadmill, LE8710 M, Bioseb) with a slope of 5°. During a warm-up phase, the speed of the treadmill was progressively increased every 2 min for 10 min (5–25 cm/s). This phase was followed by an acute exercise phase where the speed of the treadmill was increased by 5 cm/s every 15 min (30–40 cm/s) until the mice showed signs of exhaustion. The rear of the treadmill was equipped with a low-voltage electric stimulating bar to encourage each mouse to run. The bar was set to deliver 0.2 mA at a frequency of 0.25 Hz, which caused an uncomfortable shock but did not injure the animal. Number of shocks was recorded, and the electric delivery was stopped if 50 shocks were reached. The mice were previously familiarized with the tests 1 week before the evaluation.

### Skeletal Muscle Strength Evaluation

The strength of upper limbs was measured using a grip test equipped with a bar (Bio-GS3, Bioseb). After three measurements, the best value was recorded and the maximal strength was expressed in Newton per gram (N/g).

### Body Composition

Lean and fat masses were measured using an NMR Benchtop System (Minispec, Bruker France SAS). This instrument, which uses low-frequency (7.5 MHz) nuclear magnetic resonance, provides non-invasive examination of living animals such as mice with reduced animal stress, allowing measurements of fat tissue, lean tissue, and free fluid composition.

### Skeletal Muscle Dissociation

Of each TLA (injured and control), 3/4 were washed with phosphate-buffered saline (PBS), minced, and digested with 2 mg/ml of type A collagenase in 1.5 ml of DMEM medium supplemented with 10% fetal bovine serum (FBS) for 60 min at 37°C. The muscle was further dissociated by performing five passages through a 3-ml syringe with an 18 G needle. After an additional 15 min of digestion and a second round in the syringe, the homogenate was diluted three times in DMEM (10% FBS), filtered (70 μm filter), and centrifuged at 300 × *g* for 20 min. The cell pellet was resuspended in PBS supplemented with 0.5% FBS at a final concentration of 5 × 10^6^ cells/ml.

### Fatty Acid Oxidation Assay of CD4^+^ T Cells

Spleen and lymph nodes of control and KO-T PPARβ/δ mice were processed (either using a gentle MACS Dissociator and appropriate gentle MACS C tubes provided by the manufacturer (Miltenyi Biotec) for spleen, or pestle and mortar for lymph nodes), in order to obtain cell suspensions in PBS, pH 7.2, containing 0.5% FBS and 2 mM EDTA. Cell suspensions from both organs were pooled before continuing with the isolation of CD4^+^ cells by magnetic labeling and separation using CD4 (L3T4) microbeads and LS or MS columns, respectively, following the manufacturer protocols (Miltenyi Biotec). The isolated CD4^+^ cells were cultured at a concentration of 4 × 10^5^ cells/well in a 48-well plate in RPMI containing 10% FCS, 100 units/ml penicillin/streptomycin, and 50 μM 2-mercaptoethanol. Cells were activated with anti-CD3/anti-CD28 beads (Dynabeads mouse T-activator CD3/CD28, Invitrogen) following instructions provided by the manufacturer. For certain conditions, 50 μM etomoxir (Sigma) was added as well. After 48 h, media were refreshed and 10 μl/well of a mix of radioactive and non-radioactive palmitate coupled to BSA (2:1 ratio; 15 μM fatty acid-free BSA (Sigma), 30 μM Na-palmitate (Sigma), and 10 μCi [0.83 μM [^9,10^**]**-3H-palmitic acid (Perkin Elmer)] was added to each well. The radioactive and non-radioactive palmitate was coupled to BSA by first quickly adding the non-radioactive palmitate preheated at 70°C to BSA preheated at 50°C, followed by addition of the radioactive palmitate at this mix at 50°C. After a 24-h additional incubation, 100% trichloroacetic acid (10% final) was added to the cell suspensions and proteins were allowed to precipitate. After centrifugation, NaOH (final concentration 0.75 M) was added to the supernatant to increase pH to 12. Subsequently, 400 μl of supernatant was applied to ion-exchange columns (Dowex 1 × 8–200, Sigma), and ^3^H_2_O was recovered by eluting with 2.5 ml of H_2_O. A 0.75-ml aliquot was then used for scintillation counting. Results were expressed as counts per minute (CPM) per 10^6^ cells.

### Cell Preparation and Flow Cytometry Analysis

All flow cytometry staining steps were performed at 4°C in the dark. Cell suspensions obtained as described above were incubated with fluorescently labeled primary antibodies for 20 min in PBS, 0.5% FBS, and 2 mM EDTA (FACS buffer). The following antibodies from eBioscience were used to analyze T cell populations in the thymus: CD3-fluorescein isothiocyanate, CD3-phycoerythrin, CD4-allophycocyanin, TCRβ-phycoerythrin-Cy7, TCRγδ-phycoerythrin, CD44-phycoerythrin-Cy7, CD62L-fluorescein isothiocyanate, CD25-phycoerythrin, and CD44-phycoerythrin. CD8-Peridinin chlorophyll antibody and Fc Block (antimouse CD16/CD32 monoclonal antibody) were from BD Biosciences. The following antibodies from Miltenyi were used to analyze T cell populations in lymph nodes and in SKM: CD3-fluorescein isothiocyanate, CD4-allophycocyanin-vio770, CD25-phycoerythrin, and FoxP3-allophycocyanin. After cell surface staining, cells were washed with FACS buffer. For intracellular staining of FoxP3, “FoxP3 staining buffer set” from Miltenyi was used according to manufacturer’s protocol. Briefly, cells were incubated in 1× fixation/permeabilization solution for 30 min. Cells were then washed with FACS buffer, centrifuged, and resuspended in 1× perm solution containing FoxP3-APC antibody. After 30 min, cells were washed with 1× perm solution, centrifuged, and resuspended in FACS buffer. Flow cytometry acquisition was performed using a BD FACSCanto II flow cytometer (BD Biosciences), and data analyzed using FlowJo software.

### RNA Extraction and Quantitative Real-Time PCR

Total RNA was extracted from cells or tissues with Trizol reagent (Invitrogen) and 1 μg of RNA was reverse-transcribed using a QuantiTect Reverse Transcription Kit (Qiagen) on a QcyclerII. Quantitative PCR was done using SYBR Premix Ex Taq (Tli RNase H Plus) (Ozyme) on a StepOne machine (Life Technologies). The mRNA levels of all genes were normalized to 36B4 transcript levels. Sequences of primers used are as follows: *Ppar*β/δ-F: GCA-GCC-TCA-ACA-TGG-AAT-GTC; *Ppar*β/δ-R CAT-ACT-CGA-GCT-TCA-TGC-GG. These primers allow the detection of intact exon 4 (wild-type form) and does not detect the recombined transcript, deleted from this exon (mutated form). *Cpt1a*-F: CTC-AGT-GGG-AGC-GAC-TCT-TCA; *Cpt1a*-R: GGC-CTC-TGT-GGT-ACA-CGA-CAA; *36B4*-F: TCC-AGG-CTT-TGG-GCA-TCA; and *36B4*-R: CTT-TAT-CAG-CTG-CAC-ATC-ACT-CAG-A.

### Statistical Analyses

The results are presented as means ± standard deviations. All data were analyzed using Statview and GraphPad Prism v 5.0 software (San Diego, CA, United States). For each dependent variable under consideration, and according to assumptions for statistical analysis (i.e., normal distribution, equal variance), we performed: (1) non-parametric Mann–Whitney *U*-test to investigate the effect of genotype in young animals; (2) two-way ANOVA analyses to investigate independent effects of genotype and aging and the interaction effects between genotype and aging; (3) two-way ANOVA analyses to investigate the interaction effect between SKM injury (according to time of killing) and genotype. For this last analysis, the contralateral uninjured leg was used as control. We verified that no difference was shown according to gender of mice. This was not the case except for thymus, spleen, and VAT mass. For analysis of this variable, gender was taken into account as an additional factor in ANOVA analysis to isolate independent effect of genotype, aging and interaction effect between genotype and aging. Fisher PLSD and Newman-Keuls *post hoc* tests analyses were performed for multiple comparisons when statistical significance was reached for interaction effects. Statistical significance was accepted at *p* < 0.05.

## Results

### Specific T Cell PPARβ/δ Invalidation Had No Effect on Thymic T Cell Development but Increased CD4^+^ T Cell Prevalence in Lymph Nodes

**FIGURE 1:**
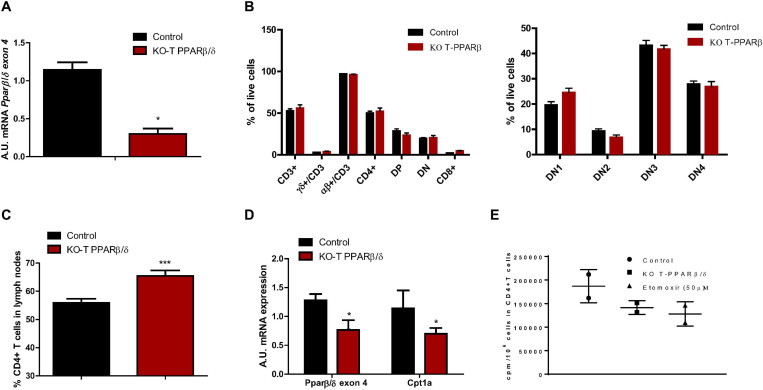
Characterization of T cells in thymus and lymph nodes of young KO-T PPARβ/δ mice compared to young control mice. **(A)** *Ppar*β/δ *exon 4 mRNA level in T cells*. Relative *Ppar*β/δ mRNA levels in isolated CD3^+^ T cells from spleen and lymph nodes. Isolation of CD3^+^ cells was performed using magnetic labeling according to the manufacturer protocol. RNA was extracted as described in “Materials and Methods”; *36B4* has been used as housekeeping mRNA; *n* = 3 in each group. **(B)** *T cell population analysis in thymus and thymic development of T cells*. Cell suspensions from thymus were obtained as described in “Materials and Methods” for cell suspensions from spleen. Left panel, percentages of the different T cell populations in thymus obtained from flow cytometry analyses are shown: CD3^+^ (and among CD3^+^, relative percentages of TCR- αβ and TCR- γδ positive T cells), CD4^+^, CD8^+^, DN (CD4^–^ CD8^–^), DP (CD4^+^CD8^+^). Right panel, relative percentages of DN1 (CD25^–^ CD44^+^), DN2 (CD25^+^ CD44^+^), DN3 (CD25^+^ CD44^–^), and DN4 (CD25^–^ CD44^–^) among DN cells are indicated (*n* = 6 par group). **(C)** *CD4*^+^ *T cell prevalence in lymph nodes in CD3*^+^ *T cells*. Cell suspensions from lymph nodes were obtained as described in “Materials and Methods.” Flow cytometry analyses were performed to analyze CD4^+^ in CD3^+^ cell gating (control, *n* = 11; KO-T PPARβ/δ, *n* = 8). **(D)** *Ppar*β/δ *exon 4 and Cpt1a mRNA expression in lymph nodes*. RNA was extracted from lymph nodes cell suspension as described in “Materials and Methods.” The relative amounts of *Ppar*β/δ *and Cpt1a* mRNA were quantified using *36b4* as housekeeping mRNA (control, *n* = 3; KO-T PPARβ/δ, *n* = 5). **(E)** *Palmitate oxidation of isolated CD4*^+^ *T cells*. FAO was measured as ^3^H-palmitate conversion to ^3^H_2_O and quantified as CPM/10^6^ cells in *in vitro*-activated CD4^+^ cells treated or not with 50 μM etomoxir (*n* = 2 per group). Values are presented as box and whiskers min to max. Except for **(E)**, values are mean ± SD. Statistical analyses were performed using Mann–Whitney *U*-test.*p < 0.05, different from control mice.

We used a mouse model invalidated for PPARβ/δ specifically in T cells (KO-T PPARβ/δ) and verified that non-recombined *Ppard* mRNA levels (still containing exon 4) were lower in CD3^+^ T cells from spleen and LNs of young KO-T PPARβ/δ mice compared with young control mice (Figure 1A). We would like to point out that the remaining non-recombined *Ppard* transcript levels measured in the knockout cells were only detected after an average of 34 PCR cycles, if at all, suggesting that remaining PPARβ/δ expression was non-existent to very low (Nadra et al., 2006). Thymus weight (Table 1) and cell count (data not shown) were not different in KO-T PPARβ/δ mice compared with the control mice. Thymic T cell development was not altered in KO-T PPARβ/δ mice. Indeed, cytometric analyses of isolated thymic T cells showed that young KO-T PPARβ/δ mice exhibit similar CD3^+^ T cells percentage and double negative (DN) subsets prevalence, compared with the control animals (Figure 1B). Flow cytometry analysis performed on isolated T cells from LNs showed that the percentage of CD4^+^ T cells in LNs was significantly higher in young KO-T PPARβ/δ mice compared with the control animals (Figure 1C). mRNAs of *Ppard*β/δ and *Cpt1a*, the main PPARβ/δ target-gene involved in FAO in lymphoid tissues (Mothe-Satney et al., 2016) were decreased in LNs of KO-T PPARβ/δ mice (Figure 1D). In addition, treatment of purified T cells from KO-T PPARβ/δ mice with a specific PPARβ/δ agonist (GW0742) was not able to induce *Cpt1a* expression, confirming loss of PPARβ/δ function. This result is part of an upcoming manuscript regarding a role for PPARβ/δ in Treg polarization. Moreover, palmitate oxidation by lymphoid organ-isolated CD4^+^ T cells from KO-T PPARβ/δ mice tended to be lower than those from control mice. Interestingly, this level of oxidation was comparable with the one observed in CD4^+^ T cells from control animals treated with etomoxir, an inhibitor of Cpt1a (O’Connor and Milone, 2020; Figure 1E).

**TABLE 1:** Effects of age and genotype on body composition.

	**Control Cre (*n* = 28)**		**KO T PPARβ /δ (*n* = 27)**		Genotype*age
	**Young mice**	**Old mice**	**Young mice**	**Old mice**	
Body weight (g)	22.7 ± 4.4	30.1 ± 5.8*	22.4 ± 2.6	27.3 ± 3.9*	n.s.
Lean mass (g)	15.3 ± 2.8	16.6 ± 1.7*	15.0 ± 1.9	18.4 ± 3.2*	n.s.
Fat mass (g)	4.4 ± 1.3	8.1 ± 3.9*	4.7 ± 1.0	5.5 ± 1.4*	*F* = 8.3; *p* = 0.006
Thymus (mg/g)	1.9 ± 0.6	1.1 ± 0.2*	1.7 ± 0.7	1.0 ± 3.0*	n.s.
Spleen (mg/g)	3.9 ± 0.8	3.5 ± 0.9	3.0 ± 0.9	3.8 ± 1.0	*F* = 9.2; *p* = 0.004
Heart (mg/g)	5.2 ± 0.6	4.6 ± 0.5	5.5 ± 0.5^§^	5.8 ± 0.8§	*F* = 6.8; *p* = 0.010
TLA (mg/g)	1.7 ± 0.17	1.42 ± 0.2*	1.93 ± 0.3^§^	1.58 ± 0.3*^§^	n.s.
Liver (mg/g)	43 ± 19	44 ± 7	49 ± 4	45 ± 6	n.s.
VAT (mg/g)	11.2 ± 0.6	24.9 ± 1.28*	13.3 ± 0.4^§^	13.2 ± 0.5^§^	*F* = 16.6; *p* = 0.0002
BAT (mg/g)	3.8 ± 0.2	4.8 ± 0.4*	3.9 ± 0.11	4.8 ± 0.2*	n.s.

Statistical analysis was performed using a two-way (age and genotype) analysis of variance (ANOVA). *p < 0.05, age effect; ^§^*p* < 0.05, genotype effect. Data are expressed as the mean ± SD in each group. Young control (*n* = 21); young KO-T PPARβ/δ (*n* = 15); older control (*n* = 7); older KO-T PPARβ/δ (*n* = 12). TLA, tibialis longitudinus anterior skeletal muscle; BAT, brown adipose tissue; VAT, visceral adipose tissue.

### Increased Accumulation of CD4^+^ T Cells in Skeletal Muscle in the Early Stage of Regeneration in KO-T PPARβ/δ Mice

**FIGURE 2:**
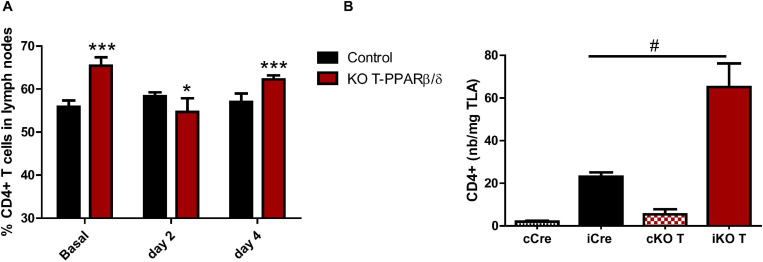
CD4^+^ T cell prevalence in lymph nodes and in TLA after cardiotoxin-induced injury of young KO-T PPARβ/δ and control mice. **(A)** *CD4*^+^ *T cell prevalence in lymph nodes*. Percentages of CD4^+^ T cells among CD3^+^ cell population in basal state and at day 2 (*n* = 5 per group) and day 4 (control, *n* = 8; KO-T PPARβ/δ, *n* = 6) after injury of TLA with cardiotoxin. **(B)** *CD4*^+^ *T cell number in TLA*. Number of CD4^+^ T cells among CD3^+^ T cell population (expressed per mg of TLA) in cells from digested TLA 2 days after injury with cardiotoxin. Left TLA of each mouse was injured and the right TLA of the same mice was injected with saline (cCre: uninjured TLA (right) of control Cre mice; iCre: injured TLA (left) of control Cre mice; cKO T: uninjured TLA (right) of KO-T PPARβ/δ mice; iKO T: injured TLA (left) of KO-T PPARβ/δ mice). Values are mean ± SD. Statistical analysis was performed using two-way (time post-injury and genotype) analysis of variance (ANOVA) test. Interaction effect between time post-injury and genotype was significant.*p < 0.05, ***p < 0.0005 different from control mice at the same time (basal, day 2, day 4); ^#^*p* < 0.05 interaction effect between genotype and injury.

We induced a myonecrosis with CTX injection in TLA to cause an immune challenge which is known to be accompanied by an accumulation of CD4^+^ T cells in injured SKM (Burzyn et al., 2013). At days 2 and 4 after the acute injury (i.e., the early stage of the inflammatory process), we collected LNs and digested TLA [injured and uninjured (control)]. As naïve T lymphocytes circulate through the lymph-vascular system and enter and exit lymphoid organs, at this early stage of the inflammatory process, we investigated whether both the proportion of LN-resident CD4^+^ T cells and the number of CD4^+^ T cells in SKM were altered in young KO-T PPARβ/δ mice. Regarding the prevalence of CD4^+^ T cells in LNs, the two-way analysis of variance ANOVA test (injury, genotype) showed a significant interaction effect (*p* < 0.0001) between the injury time and the genotype of mice (Figure 2A). As described before, the percentage of LN CD4^+^ T cells at baseline was significantly higher in KO-T PPARβ/δ compared with the control animals. However, this percentage was significantly lower at day 2 post-injury while it returned to baseline at day 4 in KO-T PPARβ/δ mice. In control mice, this percentage of CD4^+^ T cells did not significantly vary with injury. This showed a transient mobilization of CD4^+^ T cells in response to this challenge only in KO-T PPARβ/δ mice (Figure 2A). In order to investigate the concomitant difference of the presence of CD4^+^ T cells in the injured TLA at day 2 according to the genotype of mice, we retrieved the stromal vascular fraction which was then characterized by flow cytometry. At day 2, CD4^+^ T cell number (per mg of TLA) was significantly higher in injured TLA compared with uninjured TLA. Flow cytometry analysis showed that relatively few T lymphocytes were detected in uninjured TLA. The difference of their number was not significant between KO-T PPARβ/δ and control mice (Figure 2B). In contrast, in injured TLA muscle, CD4^+^ T cell number increased with a significantly higher magnitude in KO-T PPARβ/δ mice compared with the control mice (Figure 2B). The CD3^+^CD8^+^ T cells increase was not different between groups (data not shown). These results show that in young mice, invalidation of PPARβ/δ in T cells modifies both the prevalence of CD4^+^ in LNs and their accumulation in injured SKM.

### Invalidation of PPARβ/δ in T Cells Decreased the Age-Related Sequestration of Tregs in Lymph Nodes

**FIGURE 3:**
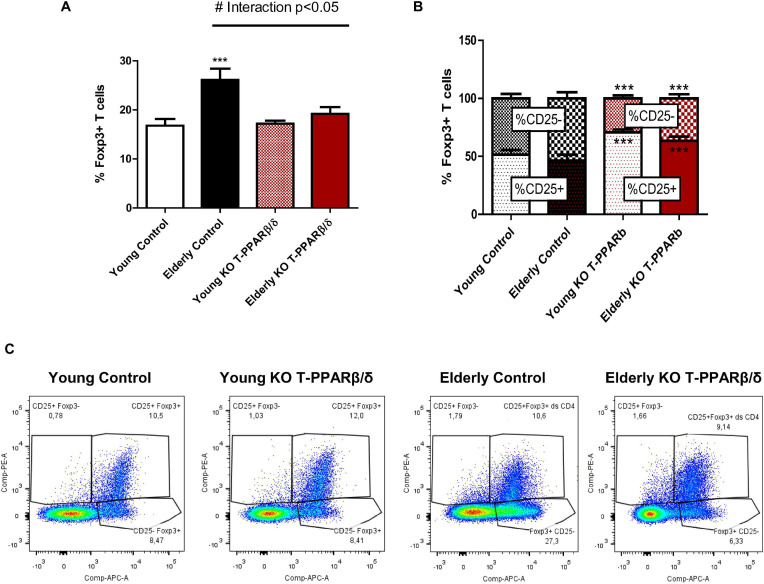
Invalidation of PPARβ/δ in T cells preserves prevalence of FoxP3^+^ Tregs in lymph nodes with age. Percentage of **(A)** labeled FoxP3^+^ Tregs in CD3^+^CD4^+^ T and **(B)** of the subpopulations CD4^+^CD25^–^ and CD4^+^CD25^+^ of FoxP3^+^ Tregs cells, in young and older mice invalidated for PPARβ/δ in T cells (KO-T PPARβ/δ) or not (control). **(C)** Representative flow cytometry dot plots of CD4^+^CD25^+^FOXP3^+^ cells and CD4^+^CD25^–^ FOXP3^+^ cells among the CD3^+^CD4^+^ T cells in lymph nodes of all mice. Values are mean ± SD. Statistical analysis was performed using a two-way (age and genotype) analysis of variance (ANOVA). ^#^*p* < 0.05, interaction effect between age and genotype; ***p < 0.005, different from age-matched control mice. Young control (*n* = 11); young KO-T PPARβ/δ (*n* = 8); older control (*n* = 7); Older KO-T PPARβ/δ (*n* = 12).

Among CD4^+^ T cells, the population of Tregs is the highest mobilized in challenged SKM (Burzyn et al., 2013) but to a smaller extent with aging (Kuswanto et al., 2016). As aging has been repeatedly shown to increase CD4^+^ Treg population sequestration in LNs (Nikolich-Zugich, 2014) and knowing that a majority of CD4^+^ Tregs in LNs are resident and not circulating T cells in old mice (Durand et al., 2018), we next investigated whether invalidation of PPARβ/δ in T cells modified the prevalence of Tregs in LNs of both young and older animals. The transcription factor FoxP3 is essential for specifying the lineage and immune suppressive function of Tregs (Fontenot et al., 2003). No effect of the genotype was evidenced in young mice on the prevalence (Figure 3A) or mean intensity fluorescence (MFI) (not shown) of FoxP3^+^ T cells in LNs. Older control mice had a significantly higher percentage of FoxP3^+^CD4^+^-labeled T cells in LNs (Figure 3A), but no difference was observed for MFI (not shown). However, as evidenced by the two-way ANOVA test, a significant interaction effect between the two factors “aging” and “genotype” was shown. In older KO-T PPARβ/δ mice, the prevalence of FoxP3^+^CD4^+^ T cell was not different from those characterized in young mice (Figure 3A). Tregs constitutively express CD25, the α subunit of the IL-2 receptor. IL-2 signaling is critical for maintaining the homeostasis and competitive fitness of Tregs (Fontenot et al., 2003). The prevalence of CD25^–^ T cells among CD4^+^FoxP3^+^ T cells has been shown to be higher in aged animals compared with young animals, and this population has been shown to become functionally heterogeneous with aging (Nishioka et al., 2006). CD25 staining of FoxP3^+^ T cells was performed to differentiate FoxP3^+^CD25^–^ and FoxP3^+^CD25^+^ subpopulations. An independent increase with age was shown by two-way ANOVA analysis for FoxP3^+^CD25^–^ cell prevalence. Moreover, a significant independent genotype effect was shown for both prevalence of FoxP3^+^CD25^–^ and FoxP3^+^CD25^+^ which were, respectively, lower and higher in KO-T PPARβ/δ animals (Figure 3B). FoxP3^+^CD25^–^ population was significantly lower in older KO-T PPARβ/δ compared with age-matched control mice (Figures 3B,C).

Together, our results show a preventive effect of the invalidation of PPARβ/δ in T cells against age-induced increase in Tregs prevalence in LNs, with a preservation of the population of FoxP3^+^CD25^+^ cells.

### The Invalidation of PPARβ/δ in T Cells Prevented the Age-Related Change of Body Composition and the Loss of Endurance Capacity

**FIGURE 4:**
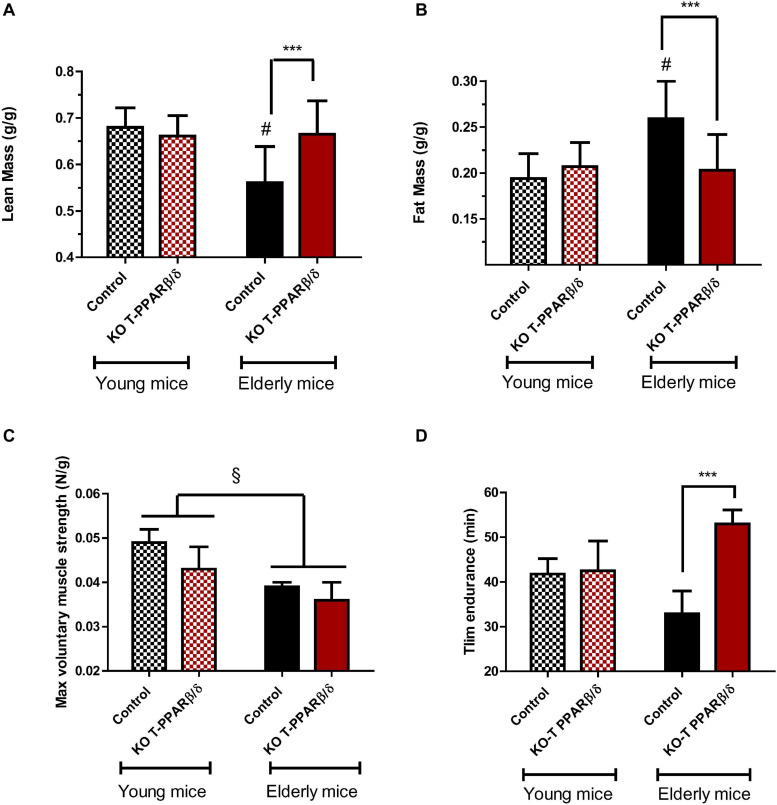
Invalidation of PPARβ/δ in T cells preserves body composition and endurance capacities with age. Comparison of **(A)** lean and **(B)** fat mass, per weight of mice, according to age (young: 12–16 weeks, striped bars; older; 39–45 weeks, full bars) and genotype. Effect of age and genotype on **(C)** baseline maximal voluntary grip strength (*N* per g of body weight) and on **(D)** exhaustive treadmill running time (Tlim). Values are mean ± SD. Statistical analysis was performed using a two-way (age and genotype) analysis of variance (ANOVA). ^#^*p* < 0.05, different from control young mice; ***p < 0.005, different from age-matched control mice. ^§^ *p* < 0.05, different from young animals. Young control (*n* = 11); young KO-T PPARβ/δ (*n* = 8); older control (*n* = 7); older KO-T PPARβ/δ (*n* = 12).

Aging is characterized by a progressive deterioration of physiological systems, and the loss of muscle mass is one of the most recognizable, leading to muscle weakness and mobility impairments. The link between immune change with aging and muscle mass maintenance is not clear. We basically asked ourselves the question of whether the invalidation of PPARβ/δ specifically in T cells would have repercussion on body composition and exercise capacities in both young and older mice. We performed NMR analysis of body composition. Lean mass was significantly lower (Figure 4A and Table 1), and body mass and fat mass were significantly higher in older control mice compared with young animals (Figure 4B and Table 1). Interaction effects between genotype and aging were significant for both lean and fat mass but not for body weight. Indeed, older KO-T PPARβ/δ mice had significant higher lean mass (Figure 4A) and lower fat mass (Figure 4B) compared with their older control counterparts. Strikingly, lean and adipose tissue masses of older KO-T PPARβ/δ mice were not different from those of young animals (Figure 4B).

Next, tissues that were taken during the killing were weighted (Table 1), and their weight normalized to the body weight of animals to correct for intermice variability. Moreover, we considered gender a covariable to avoid a confounding effect when necessary. An effect of age was observed on thymus and BAT weights which were decreased for both genotypes (Table 1). However, older KO-T PPARβ/δ mice did not exhibit the decreasing effect of age on the mass of spleen and heart and the increasing effect of age on VAT mass (Table 1). No difference between genotypes was evidenced for liver, thymus, spleen, and for BAT, but TLA SKM and heart masses were both significantly higher in KO-T PPARβ/δ mice compared with control mice (Table 1). These results suggest that the effect of genotype on lean mass is mainly due to a muscle mass maintenance.

To capture a physical capacity profile of these mice, we undertook assessments for strength and endurance analysis. Our data did not reveal marked differences according to the genotype of mice in maximal voluntary grip strength which was, however, significantly lower in older mice compared with young animals (Figure 4C). In contrast, treadmill endurance performance was significantly higher in KO-T PPARβ/δ mice (Figure 4D). The difference was two times higher than their “control” older counterparts despite that it might be underestimated, as we have stopped the test after 1 h of running as requested by the institutional ethic committee for the use of laboratory animals. This last result suggests that KO-T PPARβ/δ mice are protected against an age-related decrease in running capacity despite having never being trained.

## Discussion

PPARβ/δ is a key player in the transcriptional regulation of lipid metabolism but less well studied than other subtypes (i.e., PPARα and PPARγ) in T cells. Contrary to an activation of the pathway by its specific agonist GW0742 (Mothe-Satney et al., 2016), we showed in this study that the invalidation of PPARβ/δ specifically in T cells decreased FAO potential of CD4^+^ T cells. Moreover, KO-T PPARβ/δ mice had no significant defect in thymus cellularity and T cell development. By using a mouse model of global invalidation of PPARβ/δ, Zhao et al. (2018) showed defects in PPARβ/δ-deficient thymocytes. This apparent contradiction suggests that PPARβ/δ expressed in other cell types than T cells in the thymic environment would be mainly responsible for this phenotype. In contrast, we have previously shown that the overexpression of PPARβ/δ specifically in T cells alters T cell development, suggesting that low or no expression of this nuclear receptor is more supportive of normal T cell thymic development and proliferation (Mothe-Satney et al., 2016). Most changes in T cells seemed to occur in the periphery in KO-T PPARβ/δ mice.

One of the main consequences of PPARβ/δ activation is to increase fatty acid metabolism. Interestingly, increased FAO of CD4^+^ memory T cells—a subset of CD4^+^ T cells that are able to use FAO for their energetic metabolism—could contribute to the decline in T cell functions with age and in their ability to survive (Yanes et al., 2019). In our study, *Cpt1a* was decreased in CD4^+^ T cells and in LNs, due to the invalidation of PPARβ/δ. This decrease in *Cpt1a* seemed to induce a concomitant decrease in FAO in isolated CD4^+^ T cells and was associated with a higher prevalence of CD4^+^ T cells in LNs of young KO-T PPARβ/δ mice. Based on genetic models of Cpt1a deletion in T cells, Raud et al. (2018) have demonstrated that in the absence of Cpt1a, T effector cells, memory T cells, and Treg cell development and function occur normally. This indicates that Cpt1a is dispensable and that these cells could adapt metabolically to a reduction in long chain (LC)-FAO (Raud et al., 2018). This could also explain why the prevalence of Tregs was unchanged in LNs of young animals. However, with aging, Tregs were shown to accumulate in secondary lymphoid organs (Darrigues et al., 2018; Durand et al., 2018), but this effect of age was surprisingly not observed in KO-T PPARβ/δ mice. Effects of Cpt1a invalidation was not characterized in old animals in the study of Raud et al. (2018), but regarding our results and those from Yanes et al. (2019), it could be possible that LC-FAO would act as a determinant of the loss of T cell functions and survival with age. When further characterizing Tregs using FoxP3 and CD25 markers, we found a preserved functional FoxP3^+^CD25^+^ prevalence and a marked lower prevalence of CD25^–^ cells in LNs from older KO-T PPARβ/δ mice. It has been shown that the absence of CD25 in peripheral Tregs was determinant of their increased susceptibility to oxidative stress, mitochondrial dysfunction, and apoptosis and of the transcription of lipid and cholesterol biosynthetic key enzymes (Toomer et al., 2019). Moreover, CD25 expression at the cell surface was dependent on a redox-regulated signaling pathway (Secchi et al., 2020) and was decreased on Tregs from aged mice (Chougnet et al., 2011). Taking all these data into account, since older KO-T PPARβ/δ mice display less FoxP3^+^CD25^–^ cells (accompanied by a concomitant increase in FoxP3^+^CD25^+^ cell proportion), one might expect that Tregs of these mice are preserved from the effects of aging and oxidative stress, and thus more functional and less prone to immunosenescence. The deletion of PPARβ/δ in T cells would potentially contribute to increase the stress-related pathway responses by maintaining NF-κB activity in aged mice, which could sustain naive T cell turnover essential to maintain naive T cell diversity including Tregs (Mold et al., 2019). Moreover, NF-κB p65 subunit, which physically interacts with PPARβ/δ (Schnegg et al., 2012), was demonstrated to be crucial for the expression of FoxP3 and for mature Tregs homeostasis in secondary lymphoid organs (Oh et al., 2017). While the exact mechanisms remain to be determined, our results suggest that Tregs of KO-T PPARβ/δ mice might be protected from the negative effects observed on Tregs fate with aging. This could have implications for the immunosuppression that occurs with aging.

PPARβ/δ drives redox and metabolic changes in T cells that could modify some of their properties such as their capacity to be mobilized in tissues. Recent data from the group of D. Mathis have shown that CD4^+^ T cells present in injured mouse SKM are mainly Tregs, constituting 40 to 60% of the CD4^+^ T cell compartment (Burzyn et al., 2013; Kuswanto et al., 2016). Interestingly, when we induced SKM injury to study local acute immune response, we observed an increase in the number of infiltrated CD3^+^CD4^+^ T cells in injured TLA of KO-T PPARβ/δ mice compared with control mice at day 2 post-injury. As the number of resident CD4^+^ T cells remained unchanged in the uninjured TLA in both groups of mice, our data suggest an increase in their recruitment upon injury. This hypothesis is supported by a higher capacity of CD4^+^ T cells of KO-T PPARβ/δ mice to be mobilized from lymph nodes, as suggested by the increase in CD4^+^ T cells within SKM concomitant with a decrease of CD4^+^ T cells in LN of KO-T PPARβ/δ mice. Observation of an increase in Tregs in injured SKM is generally made at day 4, when they reached a peak. The kinetic is quite similar to that of anti-inflammatory M2 macrophages (Burzyn et al., 2013; Kuswanto et al., 2016; Tidball, 2017). However, little is known about the type of CD3^+^ T cells in injured SKM at earlier time points (day 2) corresponding to the peak of proinflammatory M1 macrophages. Immune cells are important sources of cytokines that can affect myogenesis and regulate SKM growth and regeneration (Fu et al., 2015; Tidball, 2017; Wang et al., 2018). We do not know if the infiltrated cells had improved the regeneration process itself but importantly, we show in this study that lean mass of KO-T PPARβ/δ mice was maintained with age. This lean mass maintenance was mainly related to a higher muscle mass (SKM and heart) compared with age-matched control animals. We recently obtained data (unpublished) showing that long-term treatment with GW0742 had a negative effect on SKM maintenance (mainly oxidative SKM). These results suggest that chronically activating PPARβ/δ pathway could be negative for the maintenance of SKM integrity, an effect which differed *a priori* from the short-term “exercise-mimetic” effect of its activation (Narkar et al., 2008).

It has been shown that many features of physiological aging (including sarcopenia) and the main features of immunosenescence, could be partly prevented by maintaining a high level of physical activity during life (Pollock et al., 2015; Duggal et al., 2018). Our older KO-T PPARβ/δ mice, similarly to young mice, maintained a surprising high running endurance. Normally, for a fixed running speed, oxygen consumption and duration of running decrease with aging in mice (Schefer and Talan, 1996). Furthermore, at exhaustion, the capacity to run of older KO-T PPARβ/δ mice was progressively declined, similarly to young animals, whereas older control mice refused to run as was previously reported (Schefer and Talan, 1996). Relationships between endurance performance and body weight or with the SKM oxidative adaptation marker citrate synthase, were not clearly evidenced in our study (Supplementary Figure 1), suggesting that exhaustion time in our mice is not principally related to these parameters. Exhaustion time is mainly a reflection of fatigue which is among the most common presenting complaints in older adults (Simonsick et al., 2016). Performance capacities and perceived fatigability—the two suggested attributes of fatigue—are modulated during exercise by multiple factors such as contractile capacities, motivation, core temperature, and pain (Enoka and Duchateau, 2016) which were not controlled in our study. It is clear that modification in T cells (i.e., PPARβ/δ deficiency) might play a role in a lifetime exercise-trained phenotype, but further research needs to be done to provide definite proof that T cell modulation affects endurance and to unravel the mechanisms by which it can do so.

Together, our data link modification of T cells to body composition and running performance. Even though we are aware of the various limitations of this study, we think that these surprising results lead to novel hypotheses for reducing age-related changes in muscle by manipulating T cells. Mechanisms remain to be elucidated but many questions have emerged from this study. Did T cell secretome of KO-T PPARβ/δ mice lead to a rejuvenating secretome able to maintain immune system and body composition into old age? Did the sensitivity to stress or cytokines that are known to be involved in exercise adaptations increase? Did the expression of immune modulatory surface molecules change? Did the modulation of energetic substrates used create a nutritional environment favorable for the maintenance of physical capacity with age? Our study is the first to show that the modification of a transcriptional regulator of fatty acid metabolism in T cells could have a positive impact on the preservation of body composition and the capacity to run with aging despite an *a priori* sedentary behavior of mice. Among all the characteristics of mice advancing with age, we come to a rather striking set of observations leading us to believe that the invalidation of PPARβ/δ in T cells protects them from pathological aging, rendering these mice protected from susceptibility to age-related immune changes and frailty.

## Data Availability Statement

The raw data supporting the conclusions of this article will be made available by the authors, without undue reservation.

## Ethics Statement

The animal study was reviewed and approved by Comité Institutionnel d‘Éthique Pour l‘Animal de Laboratoire (CIEPAL)-AZUR n° 28; N° 2018110914193037.

## Author Contributions

A-SR and IM-S designed the research. A-SR and IM-S conducted research and analyzed data. WW provided B6.Ppard^TM 1*Mtz*^ mice. JM, JN, BS, SL, and GL assisted with the experiments. A-SR and IM-S wrote the manuscript. JN, GC, BS, GL, and WW reviewed and edited the manuscript. All authors read and approved the final manuscript. The authors report no conflicts of interest.

## Conflict of Interest

The authors declare that the research was conducted in the absence of any commercial or financial relationships that could be construed as a potential conflict of interest.
